# Parenting stress spirals and a gendered labour division over the Christmas holidays

**DOI:** 10.1038/s44271-026-00535-3

**Published:** 2026-10-24

**Authors:** Elouise Botes, Julia Reiter, Samuel Greiff, Ziwen Teuber

**Affiliations:** 1 Cognitive Science and Assessment Institute, University of Luxembourg, Esch-sur-Alzette, Luxembourg; 2 Luxembourg Center for Educational Testing, University of Luxembourg, Esch-sur-Alzette, Luxembourg; 3 Department of Occupational, Economic and Social Psychology, University of Vienna, Vienna, Austria; 4 Technical University of Munich, Munich, Germany

**Keywords:** Human behaviour, Psychology

## Abstract

The Christmas period heightens parental demands and invisible labour, yet little is known about how these processes unfold in daily life. This study used experience sampling data from 293 UK parents over 35 days to examine temporal associations between cognitive labour, emotion suppression, and subjective stress across the holiday season. Dynamic Structural Equation Modelling revealed strong autoregressive stability for all constructs and reciprocal moment-to-moment links between cognitive labour and stress, indicating a self-reinforcing cycle. Cognitive labour and stress also predicted subsequent emotion suppression, suggesting that emotion suppression operates as a situational coping response to mental and stress overload. At the between-person level, social support reduced emotion suppression but did not buffer stress or cognitive labour. Gender differences emerged only for cognitive labour, with mothers reporting higher levels than fathers. Findings highlight the dynamic interplay between invisible labour and stress and underscore the psychological strains parents face during the holidays.

## Introduction

Santa Claus is coming to town—but in the meantime, Mrs. Claus has to make the magic of Christmas happen by organising activities, buying presents, managing the household and childcare, and providing emotional support. Household labour is commonly divided into physical, cognitive and emotional labour^1,2^, and its division between parents is a common source of relationship strain and personal stress. In Western countries, the Christmas period is a prime example of a time period during which household labour is doubly exacerbated: firstly, special occasions like holidays are tied to cultural expectations involving household labour. Secondly, cultural holidays like Christmas typically come with a period of school holidays, meaning the daily routines, including out-of-home childcare is disrupted. During this period, parents are often expected to organise multiple family gatherings, coordinate travel plans to grandparents or other extended family, purchase and wrap gifts, prepare traditional meals, maintain family traditions such as decorating the Christmas tree, and manage children’s expectations surrounding the holiday season. These obligations frequently require substantial cognitive planning and emotional regulation, such as concealing stress, frustration or interpersonal tensions while maintaining a festive and positive atmosphere for children and extended family members.

Parenting involves multiple forms of labour, ranging from the visible tasks of childcare and housework to less tangible but equally demanding forms of invisible labour^3^. Among these, cognitive labour comprises the constant anticipation and coordination of tasks, from scheduling medical appointments to ensuring holiday preparations run smoothly. Its unbounded and never-ending character means that it intrudes into rest and leisure, contributing to chronic stress and reduced psychological well-being. However, these effects are not evenly distributed as mothers typically shoulder a disproportionate share of invisible labour^3^, while contextual factors such as socio-economic status (SES), single parenthood, and levels of social support further shape stress outcomes^4–6^.

In addition to cognitive labour, the demands of parenting may also require parents to regulate and suppress their emotions in interactions with their children. Parenting norms, particularly in Western countries, often encourage parents to suppress irritation, frustration, or exhaustion while continuing to display warmth, patience, and enthusiasm towards their children^7^. This process of emotion suppression aligns with Gross’s conceptualisation of expressive suppression as an emotion regulation strategy involving the inhibition of outward emotional expression^8^. At the same time, it overlaps conceptually with the surface acting component of emotional labour^9^, which refers to modifying or masking emotional displays in order to meet social expectations^10^. In parenting contexts, such emotion suppression may come at a substantial psychological cost, as suppressing negative emotions while displaying positive ones has been associated with stress, burnout, emotional distancing, and reduced responsiveness to children’s needs^7,11^.

Most prior studies have used cross-sectional or qualitative methods^12,13^, limiting understanding of how parenting demands and stress interact over time. The present study addresses this gap by employing experience sampling methodology to capture daily fluctuations in emotion suppression, cognitive labour, and stress across the Christmas holidays, a period when invisible parental demands are both intensified and unavoidable. By examining reciprocal effects within parents’ everyday experiences, and considering gender and SES, this study provides a dynamic account of how cognitive and emotional demands are associated with parental well-being. Furthermore, this study includes fathers as well as mothers, allowing for a comparison of their experiences where previous studies were largely focused on mothers alone.

## Emotional and cognitive demands of parenting

The broader literature on household labour, invisible labour, and mental load frequently encompasses both the cognitive and emotional demands associated with parenting and family management^12,14^. Within this literature, the cognitive demands of parenting are commonly conceptualised as cognitive labour or mental load, referring to the ongoing anticipation, planning, organisation and monitoring required to manage family life^15^. In parallel, the emotional demands of parenting have been conceptualised in multiple but overlapping ways, including emotional labour, emotion suppression, emotional fatigue and emotion regulation within caregiving contexts^16–19^. These emotional demands often involve suppressing frustration, irritation, or exhaustion while continuing to display warmth, patience and positivity towards children.

While emotional and cognitive demands of parenting are often examined independently within the literature, several studies have included both constructs under umbrella labels such as mental load^16^, household management^12^ and family load^14^. On a trait level, the cognitive and emotional demands of parenthood are moderately positively related^12^. On a state level, Barigozzi et al.^16^ utilised time-use diaries to capture cognitive labour and emotional fatigue of parents and found evidence of spill-over effects. Furthermore, greater household labour is also associated with increased stress and depressive symptoms^12,20^. These experiences of parental stress are further exacerbated by low SES, single parenthood^4,5^, gender (with mothers experiencing greater levels of parental stress in comparison to fathers^6^), and low social support^21^.

### Emotion suppression

Emotion suppression, or expressive suppression, refers to an emotion regulation strategy in which individuals inhibit or conceal the outward expression of their emotions^8^. Within Gross’s process model of emotion regulation, emotion regulation is seen as 'the processes by which individuals influence the emotions they have, when they have them, and how they experience and express these emotions'^22^ (p. 275). Expressive suppression is conceptualised as a response-focused strategy, meaning that it occurs after an emotional response has already been generated and involves regulating the behavioural expression of that emotion rather than altering the emotion itself. In parenting contexts, emotion suppression may involve concealing irritation, frustration, stress or exhaustion while continuing to display warmth, patience and enthusiasm towards children. Such regulation is closely tied to contemporary ideals of 'positive parenting'^7^, which involves the expectation that parents 'express the right emotions, with the right intensity, and in the right situations'^23^ (p. 246).

The concept of emotion suppression overlaps substantially with the surface acting dimension of emotional labour^9^. Emotional labour was originally defined as 'the management of feelings to create a publicly observable facial and body display'^10^ (p.7) and is conceptualised as consisting of two subfactors: surface acting and deep acting. Within emotional labour research, surface acting refers to suppressing or masking genuinely felt emotions and/or displaying emotions that are not truly experienced in order to conform to social expectations^17^. Conceptually, both emotion suppression and surface acting involve the inhibition or masking of emotional expression, and both have been examined in parenting and caregiving contexts^11,17,18,24^. In contrast, deep acting refers to attempts to genuinely modify one’s internal emotional state in order to experience the desired emotion rather than merely display it.

The belief that parents should suppress negative emotions and maintain positive emotional displays towards their children has become deeply entrenched in modern parenting norms^25^. This suppression of emotions may particularly affect mothers^3^, as emotional labour was seen as a gendered construct from its inception^10^. Emotion suppression and surface acting has been shown to require substantial physiological and cognitive effort^26–28^. Moreover, emotion suppression is linked to burnout, emotional distancing, lower emotional well-being, poorer relationship quality, increased stress and lower responsiveness to children’s needs^7,11,17^. Thus, although emotion suppression may be enacted with the intention of protecting children or maintaining positive family interactions, it may simultaneously impose substantial psychological costs on parents themselves.

### Cognitive labour

Cognitive labour refers to the 'backstage work [to the] frontstage work' of physical labour^29^. It is the work of anticipating needs, surveying different options for meeting those needs, picking one, and then monitoring throughout the process to ensure the results are satisfactory^15^. The study of cognitive labour remains hampered by measurement difficulties. Results of qualitative and quantitative studies on the subject frequently diverge, with qualitative studies showing large gender gaps in cognitive labour loads that quantitative studies fail to capture^15^. This is linked to the never-ending and unbounded nature of cognitive labour^3,30^: labour that does not have clear start and end points but rather continuously bleeds into everyday life and is performed in the background of other tasks is more difficult to accurately recall and quantify.

Cognitive labour plays a central role in understanding the mental load associated with housework and childrearing. Housework is typically unpaid and largely invisible; however, the physical tasks can in principle be made visible and can be redistributed. The same cannot be said for cognitive labour, which is permanently ongoing in the background of other tasks. This lack of boundaries and visibility of cognitive labour is inextricably linked to the mental load and stress it engenders^29,30^, as well as to its high susceptibility to gendered imbalances to the disadvantage of women^29,31,32^. Women do not only bear this burden, but are naturally expected to do so; this is attributed to cultural ideals of motherhood which involve the expectation that women lead child-centred lives and hold the labour performed by women in the house to a higher standard than that performed by men^29^.

## The current study

As such, the existing literature on invisible parental labour and stress has thus far established relationships between emotion suppression, cognitive labour, and stress in parents, with possible further effects based on gender, social support, and SES. However, the majority of studies examining the interaction between these variables were cross-sectional in nature^12^, qualitative^13^ or theoretical^3^. These approaches do not capture the temporal dynamics of how parenting labour unfolds in everyday life and how these fluctuations may impact stress. They also do not enable us to understand how the emotional and cognitive components of parental labour unfold in a relationship and how they may interact reciprocally with each other and with parental experiences of stress and burnout.

To our knowledge, only two studies have attempted to examine daily fluctuations in the invisible labour of parents: Le and Impett’s^11^ daily diary study on emotional labour and Barigozzi et al.’s^16^ diary study on cognitive labour and emotional fatigue. This highlights a significant gap in understanding how emotional and cognitive demands of parenting and stress fluctuate and interact in parent’s daily lives.

This gap is addressed by using experience sampling methodology during the Christmas season in the United Kingdom. Christmas is a time when demands on parents are both culturally heightened and practically unavoidable. While the Christmas period is a time of emotional significance for families^33^, it is also characterised by increased stress and family obligations^34–36^. This context provides an ecologically valid window with heightened parental demands and stress where the reciprocal effects between parental labour and stress can be observed on a daily basis. In addition, prior research has often focused exclusively on mothers^12,37^, reinforcing assumptions that parental labour is mainly a mother’s issue. We include both mothers and fathers to examine gendered patterns of cognitive labour, emotion suppression, and stress, in order to examine whether men and women experience these reciprocal processes differently.

The present study thus conceptualises the conditions that give way to the gendered dynamics of parental labour and stress. By adding methodologically robust evidence to the theoretical discussion, this study contributes towards understanding the underpinnings of the gendered invisible labour gap and its mental health consequences.

We therefore examine the following research questions:

1. How are parental emotion suppression, cognitive labour and subjective stress temporally associated with one another within parents over the Christmas holidays?
  - Are there differences between mothers and fathers?
2. Does greater social support before the Christmas holidays reduce emotion suppression, cognitive labour and subjective stress after the holidays?
3. Do sociodemographic variables (i.e. SES, single parenthood, the presence of special needs in children) predict emotion suppression, cognitive labour and subjective stress?

## Methods

### Transparency and openness

This study was part of a larger, preregistered project investigating parental momentary experiences during the Christmas season that was registered on 16 November 2023. The relationships examined between variables in this study was however, exploratory in nature and no exact hypotheses regarding reciprocal effects between emotion suppression, cognitive labour and parental stress were preregistered. The preregistration protocol is accessible at https://doi.org/10.17605/OSF.IO/4XGA6. Ethical approval was granted by the University of Vienna Departmental Review Board of the Department of Occupational, Economic, and Social Psychology (2023/W/021R). All participants provided informed consent and were financially compensated for their participation.

### Procedure

The study was conducted from November 2023 to January 2024, using the online data-collection service *Prolific*. Detailed information on data collection can be found in Teuber et al.^38^. Briefly, this study included a pre-survey (middle November), 35-day experience sampling surveys (November 30 to January 3) with three randomly timed daily measurements using the MindSampler app, and a post-survey (middle January), conducted before, during, and after the 2023 Christmas season. Supplement 1 provides details on participant compensation, procedure, and missing data handling.

### Sample

A total of *n* = 315 participants participated in the study. Participants who had completely missing data on the variables of interest at baseline, during the experience sampling period, or at follow-up, as well as those who reported having no children residing in the household, were excluded from the final dataset. After data cleaning, the final sample included 293 participants with a mean age of 38.16 years (SD = 6.92). Of them, 188 identified as females and 105 as males. Twenty-four participants (8.11%) reported being single parents, whereas the remaining 269 were married or in a committed relationship. Participants were recruited and analysed as individual parents rather than parent dyads; thus, mothers and fathers in the sample were not partners within the same household. On average, participants had 1.86 children (SD = 0.83) in their household, and the mean age of their children was 5.27 years (SD = 4.57). The median annual net household income was between £50,000 and £59,999, exceeding the UK’s median household income of £34,500 in 2023^39^. In the sample, 222 parents held a college or university degree or higher, and 185 were employed full-time.

### Measures

#### Pre- and post-survey measures

##### Emotion suppression (surface acting)

Emotion suppression was measured using items adapted from the surface acting subscale of the Emotional Labour Scale^40^ in both pre- and post-surveys. It included three items, such as 'I put on an act to deal with my child(ren) in an appropriate way'. Responses were given on a seven-point Likert-type scale (1 = *strongly disagree*, 7 = *strongly agree*), and mean scores were computed. Internal consistency for the total scale was high (*α* = 0.91 at pre-survey, *α* = 0.87 at post-survey).

##### Cognitive labour

Cognitive labour was assessed with single-item measures. In the pre-survey, participants responded to: 'In general, how much do you think about planning and organising tasks for family members?' In the post-survey, another single item was used: 'In this past festive/Christmas/winter holiday season, how much were you thinking about planning and organising tasks for family members?' Both items were rated on a frequency scale ranging from 1 (never) to 7 (always).

##### Subjective stress

A single item developed by Wong et al.^41^ was used to assess participants’ overall subjective stress in both pre- and post-surveys. Participants were asked to indicate the level of parenting stress they experienced during the past festive/Christmas/winter holiday season. Responses were given on a seven-point Likert-type scale, with values ranging from 1 (none at all) to 7 (an extreme amount).

##### Social support

We used the brief form of the Perceived Social Support Questionnaire by Kliem et al.^42^ to measure social support at pre-survey. This scale consisted of six items, such as 'I receive a lot of understanding and security from others'. All items were evaluated on a seven-point Likert-type scale (1 = *not true at all*, 7 = *very true*).

##### Sociodemographic variables

We collected information on participants’ gender, relationship status, socioeconomic status, and the presence of special needs in children at the pre-survey. Gender was dummy coded (1  = *mother*, 0 = *father*). The relationship status was assessed by asking participants whether they were single, married, or in a romantic relationship. Participants who reported being single were coded as 1 (single parents), whereas those who were married or in a relationship were coded as 0 (non-single parents). Socioeconomic status was estimated using participants’ total annual net household income, reported on a 13-point scale ranging from 1 (less than £10,000) to 13 (more than £150,000). Finally, the presence of special needs in children was coded as 1 = *with special needs* and 0 = *without special needs*.

#### Experience sampling measures

##### Emotion suppression

To assess momentary emotion suppression in the experience sampling surveys, we used a single adapted item from the same subscale: 'I am putting on an act to deal with my child(ren) in an appropriate way'. Responses were recorded on a 7-point Likert-type scale (1 = *not at all*, 7 = *extremely*). The intraclass correlation (ICC) showed that 58.8% of the total variance was attributed to between-person variance. The within-person reliability was *α* = 0.83, and the between-person reliability was *α* = 0.99, calculated following Nezlek^43^.

##### Cognitive labour

Momentary cognitive labour was assessed with the item 'How much are you planning/thinking about/organising tasks for family members?' Answers were given on a 7-point Likert-type scale (1 = *not at all*, 7 = *extremely*). ICC indicated that 41.7% variance was attributed to between-person variance. Using the measurement error autoregressive method for single-item scales^44^, the within-person reliability was 0.59, whereas the between-person reliability was 0.99.

##### Subjective stress

To capture momentary subjective stress in the experience sampling surveys, three items were included to address parenting, physical and emotional stress, respectively: 'Please indicate the level of parenting/physical/emotional stress you are currently experiencing'. All items were rated on a 7-point Likert-type scale (1 = *not at all*, 7 = *an extreme amount*). ICC indicated that 67% variance was attributed to between-person variance. The within-person reliability was 0.70, whereas the between-person reliability was 0.85.

### Analyses

We employed Dynamic Structural Equation Modelling (DSEM)^45,46^ in M*plus* 9^47^ to examine our research questions. DSEM integrates key features of time-series analysis, multilevel modelling, and structural equation modelling and decomposes observed values into temporal fluctuations within an individual (i.e. within-person components) and stable differences between individuals (i.e. between-person components).

Research Question 1 referred to within-person associations. We included momentary emotion suppression, cognitive labour and subjective stress at the within-person level. For each of these constructs, its value at moment *t* was regressed on its prior value at moment *t* − 1 to capture autoregressive effects, which also reflect the temporal stability of the construct. Furthermore, we estimated cross-lagged effects by regressing one construct at moment *t* on the lagged value of the other constructs at moment *t* – 1. All autoregressive and cross-lagged effects were specified as random, allowing them to vary across individuals. Furthermore, the residual variance of all three constructs was set to be random to capture individual differences in their variability of these constructs. Research Questions 1a, 2 and 3 referred to between-person differences. At the between-person level, we regressed each of emotion suppression, cognitive labour and subjective stress at post-survey on social support at pre-survey and parental gender while controlling for their values at pre-survey and time-invariant sociodemographic variables (i.e. single parenthood, income and the presence of special needs in children).

We estimated the DSEM using Bayesian methods with Markov Chain Monte Carlo (MCMC). Convergence was assessed with potential scale reduction factors (PSR), which evaluate the ratio of between-chain to within-chain variation^48^. PSR values near 1 reflect satisfactory convergence across MCMC chains, with values below 1.10 typically regarded as evidence of stochastic convergence^47,49^. In the final DSEM specification, two MCMC chains of 10,000 iterations each were run with a thinning interval of 50, yielding a total of 500,000 iterations. We applied default diffuse priors, ensuring that estimates were primarily driven by the observed data rather than prior distributions. For each parameter, the posterior median is reported as the point estimate together with its 95% credible interval (CI). Parameters with CIs that exclude zero are interpreted as different from null.

### Ethics statement

Ethical approval was granted by the University of Vienna Departmental Review Board of the Department of Occupational, Economic and Social Psychology (2023/W/021R). All participants provided informed consent and were financially compensated for their participation. All procedures were conducted in accordance with the Declaration of Helsinki.

## Results

On average, each of the 293 parents provided 49.32 repeated measures (SD = 23.06) during the experience sampling period, yielding a total of 14,451 observations. Model convergence for the DSEM was satisfactory, with PSR values for all parameters falling below 1.004 after 500,000 iterations.

### Associations of cognitive labour, emotion suppression and stress over time–within-person results (RQ1)

**Fig. 1:**
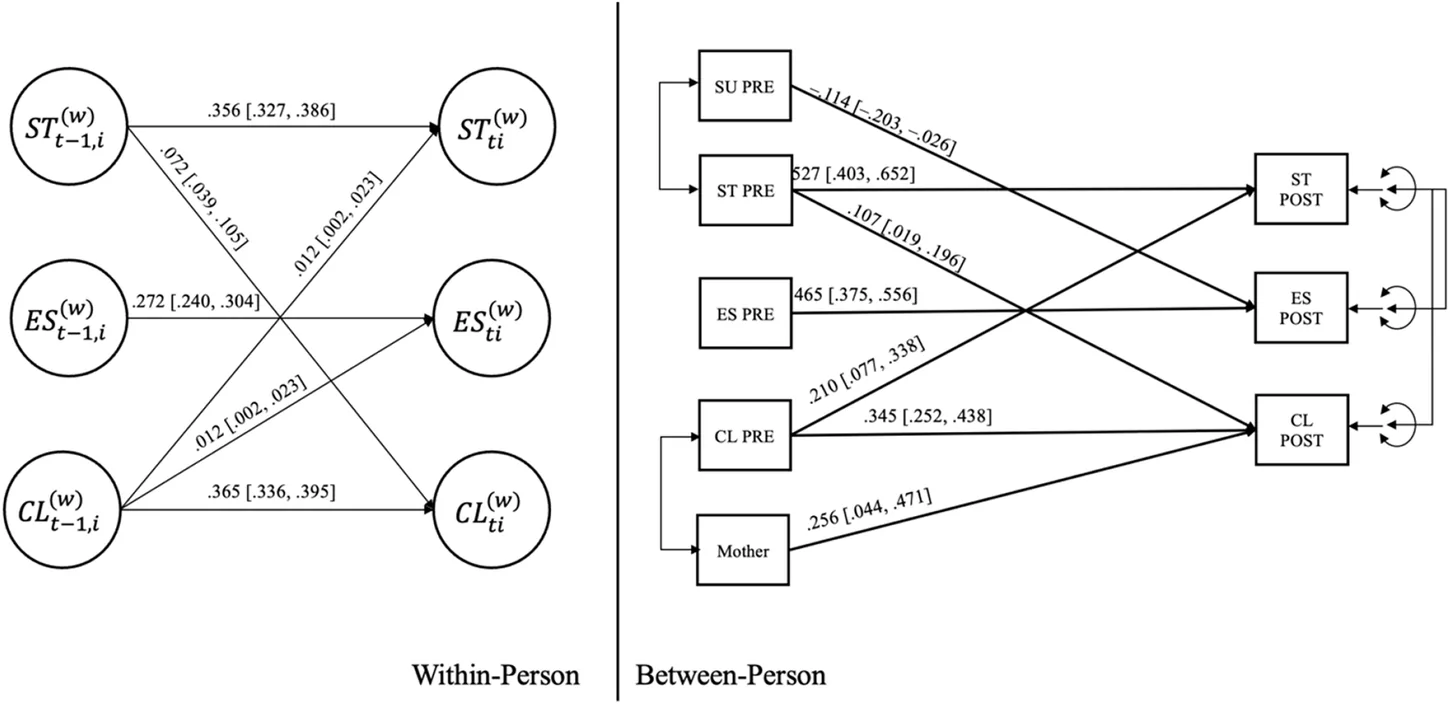
DSEM results. ST subjective stress, ES emotion suppression, CL cognitive labour, SU social support, PRE pre-survey, POST post-survey. Single parenthood, household income and the presence of special needs in children are not depicted for simplicity. *n* = 293.

First, we investigated whether and how cognitive labour, emotion suppression and stress are associated with each other throughout the Christmas period. Figure 1 illustrates the DSEM results. More detailed results are presented in Table 1.

**Table 1:** DSEM results

Parameter	Est/SE	95% CI of Est	Parameter	Est/SE	95% CI of Est
Autoregressive effects (within-person)			Cross-lagged effects (within-person)		
ST_t − 1_→ST_t_	0.356/0.015	[0.327, 0.386]	ST_t − 1_→ES_t_	0.058/0.012	[0.035, 0.084]
ES_t − 1_→ES_t_	0.272/0.016	[0.240, 0.304]	ST_t − 1_→CL_t_	0.072/0.017	[0.039, 0.105]
CL_t − 1_→CL_t_	0.365/0.015	[0.336, 0.395]	ES_t − 1_→ST_t_	0.005/0.007	[−0.009, 0.019]
Residual variances (within-person)			ES_t − 1_→CL_t_	0.009/0.012	[−0.015, 0.031]
Log_ST	−0.957/0.054	[−1.065, −0.852]	CL_t − 1_→ES_t_	0.012/0.005	[0.002, 0.023]
Log_ES	−0.597/0.101	[−0.797, −0.403]	CL_t − 1_→ST_t_	0.012/0.005	[0.002, 0.023]
Log_CL	0.092/0.063	[−0.029, 0.215]			
Regression (between-person)					
Mother→ST_POST	0.090/0.147	[−0.194, 0.384]	Mother→ES_POST	−0.087/0.140	[−0.358, 0.194]
Single→ST_POST	−0.319/0.262	[−0.854, 0.182]	Single→ES_POST	−0.420/0.241	[−0.900, 0.041]
Income→ST_POST	−0.036/0.026	[−0.087, 0.015]	Income→ES_POST	−0.012/0.025	[−0.061, 0.036]
Needs→ST_POST	−0.107/0.171	[−0.441, 0.225]	Needs→ES_POST	−0.079/0.158	[−0.382, 0.237]
ES_PRE→ST_POST	0.527/0.062	[0.403, 0.652]	ES_PRE→ES_POST	0.465/0.046	[0.375, 0.556]
CL_PRE→ST_POST	0.084/0.050	[−0.013, 0.182]	CL_PRE→ES_POST	−0.036/0.061	[−0.154, 0.086]
ST_PRE→ST_POST	0.210/0.066	[0.077, 0.338]	ST_PRE→ES_POST	0.078/0.057	[−0.037, 0.186]
SUP_PRE→ST_POST	−0.019/0.048	[−0.112, 0.076]	SUP_PRE→ES_POST	−0.114/0.045	[−0.203, −0.026]
Mother→CL_POST	0.256/0.108	[0.044, 0.471]	ES_PRE→CL_POST	0.107/0.045	[0.019, 0.196]
Single→CL_POST	−0.242/0.191	[−0.611, 0.133]	CL_PRE→CL_POST	0.345/0.047	[0.252, 0.438]
Income→CL_POST	0.008/0.020	[−0.030, 0.046]	ST_PRE→CL_POST	−0.010/0.036	[−0.083, 0.062]
Needs→CL_POST	0.095/0.124	[−0.151, 0.338]	SU_PRE→CL_POST	−0.021/0.035	[−0.089, 0.048]
Covariance (between-person)					
CL_PRE↔Income	0.156/0.186	[−0.213, 0.515]	SU_PRE↔Needs	−0.095/0.039	[−0.178, −0.021]
CL_PRE↔Needs	−0.009/0.377	[−0.067, 0.047]	Mother↔Single	0.017/0.009	[0.000, 0.034]
ST_PRE↔SU_PRE	−0.435/0.120	[−0.687, −0.211]	Mother↔Income	−0.088/0.083	[−0.254, 0.071]
ST_PRE↔Mother	0.085/0.038	[0.013, 0.162]	Mother↔Needs	0.012/0.013	[−0.013, 0.037]
ST_PRE↔Single	0.035/0.022	[−0.008, 0.078]	Single↔Income	−0.217/0.049	[−0.320, −0.127]
ST_PRE↔Income	−0.223/0.207	[−0.642, 0.184]	Single↔Needs	0.025/0.007	[0.012, 0.040]
ST_PRE↔Needs	0.070/0.032	[0.011, 0.135]	Income↔Needs	−0.166/0.070	[−0.314, −0.032]
SU_PRE↔Mother	−0.039/0.046	[−0.132, 0.048]	ES_POST↔CL_POST	0.032/0.054	[−0.070, 0.143]
SU_PRE↔Single	−0.067/0.027	[−0.122, −0.018]	ES_POST↔ST_POST	0.260/0.074	[0.122, 0.410]
SU_PRE↔Income	0.393/0.253	[−0.090, 0.891]	CL_POST↔ST_POST	0.200/0.059	[0.091, 0.323]

Residual variances were log-transformed.

*Est* unstandardized estimate, *SE* standard error, *ST* subjective stress, *ES* emotion suppression, *CL* cognitive labour, *SU* social support, *PRE* pre-survey, *POST* post-survey, *Single* single parenthood, *Needs* presence of special needs in children.

At the within-person level, we found non-null autoregressive effects for cognitive labour (*B* = 0.365 [0.336, 0.395]), emotion suppression (*B* = 0.272 [0.240, 0.304]), and subjective stress (*B* = 0.356 [0.327, 0.386]), indicating temporal stability in these constructs. In practical terms, when parents reported elevated levels on these constructs at a given moment, these effects were likely to carry over to the subsequent moment throughout the day. In addition to these autoregressive effects, we observed several cross-lagged effects. More concretely, temporal changes in cognitive labour predicted subsequent temporal changes in subjective stress (*B* = 0.012 [0.004, 0.023]), and vice versa (*B* = 0.072 [0.039, 0.105]), suggesting that when parents reported higher levels of cognitive labour or subjective stress at a given moment (relative to their own averages), they tended to experience higher levels of both at the following moment, creating a self-reinforcing downward spiral. In terms of the association between emotion suppression and subjective stress, we found a unidirectional predictive relationship: stress predicted emotion suppression (*B* = 0.058 [0.035, 0.084]) but not the other way around (*B* = 0.005 [–0.009, 0.019]). This result suggested that when parents experienced higher levels of subjective stress at a given moment (relative to their own averages), they tended to report higher levels of emotion suppression at the following moment, i.e., they suppressed their emotions in order to compensate for their stress and prevent themselves from communicating this stress to others. Finally, we identified another unidirectional cross-lagged predictive relationship between cognitive labour and emotion suppression (*B* = 0.012 [0.002, 0.023]). That is, when parents engaged in more cognitive labour than usual at one moment, they also reported suppressing emotions at the subsequent moment.

### Gendered differences in emotion suppression, cognitive labour and stress – between-person results (RQ 1a)

Mothers reported higher levels of cognitive labour at post-survey compared to fathers (*B* = 0.256 [0.044, 0.471]). No gender differences were observed for emotion suppression (*B* = − 0.087 [−0.358, 0.194]) or subjective stress (*B* = 0.099 [−0.194, 0.384]).

### Effects of social support on the holiday aftermath in terms of emotion suppression, cognitive labour and stress – between-person results (RQ2)

The second research question concerns whether greater social support at the pre-survey reduces emotion suppression, cognitive labour and subjective stress at the post-survey after controlling for their respective pre-survey levels and sociodemographics. This was tested at the between-person level in DSEM. The results showed that subjective stress at post-survey was predicted by its pre-survey level (*B* = 0.527 [0.403, 0.652]) and by cognitive labour at pre-survey (*B* = 0.210 [0.077, 0.338]), but not by emotion suppression (*B* = 0.084 [−0.013, 0.182]) or social support (*B* = − 0.019 [−0.112, 0.076]) at pre-survey after controlling for sociodemographic variables. These results indicated that parents who experienced higher stress, as well as those who more frequently engaged in cognitive labour than other parents, experienced lower stress at post-survey. Moreover, cognitive labour at post-survey was predicted by its pre-survey level (*B* = 0.345 [0.252, 0.438]) and subjective stress at pre-survey (*B* = 0.107 [0.019, 0.196]) after controlling for other variables measured at pre-survey. Thus, there was also a reciprocal association between subjective stress and cognitive labour at the between-person level. With regard to emotion suppression, we found that its post-survey levels were only predicted by its own pre-survey levels (*B* = 0.465 [0.375, 0.556]) and social support at pre-survey (*B* = − 0.114 [−0.203, −0.026]). That is, parents who received more social support at pre-survey compared to those who received less social support tended to engage in emotion suppression less frequently at post-survey.

### Effects of socio-demographic variables on emotion suppression, cognitive labour and subjective stress – between-person results (RQ 3)

The third research question examines sociodemographic differences in emotion suppression, cognitive labour and subjective stress at the post-survey, accounting for their pre-survey levels. The sociodemographic variables considered were single parenthood status, household income and the presence of special needs in children. Results indicated that none of these variables predicted the main outcomes at post-survey.

## Discussion

The division of invisible household labour and the systematic emergence of gendered imbalances therein are a critical point of tension in mixed-sex romantic relationships and in particular co-parenting relationships, as parenthood exacerbates the amount and intensity of household labour. The present study informs our understanding of the temporal dynamics of the parental cognitive and emotional household demands and sheds a light on the resulting patterns of adverse outcomes like parental stress, thereby contributing to our theoretical understanding of how these gendered dynamics unfold. We have presented data from an experience sampling study, a methodology which has thus far been under-utilised in the relevant literature, spanning the entire Christmas holiday season from a sample of around 300 UK-based parents. We used these data to investigate, firstly, how emotion suppression, cognitive labour and parental stress relate to one another over the holiday period among mothers and fathers; secondly, whether and to which extent social support helps mitigate detrimental effects of these demands; and, finally, whether there are sociodemographic effects.

### Temporal dynamics and complex interrelations: coping with and the masking of the strain of parenting demands

Across the experience-sampling period, all three constructs of cognitive labour, emotion suppression, and subjective stress showed strong autoregressive stability within parents. This indicates that parents’ moment-to-moment states were not random fluctuations but carried inertia, as elevated levels at one prompt tended to persist to the next. That is, once parents found themselves stressed, this stress was likely to continue; once they found themselves engaged in emotion suppression or cognitive labour, these activities were likely to remain ongoing. Long-term parental stress has been found to be particularly detrimental, with chronic parental stress linked to parental burnout^50^. Furthermore, parental stress has been linked to depressive symptoms, anxiety, and anger^51,52^. Thus, the stability of parental stress found during the holiday period, coupled with the known detrimental effects of chronic parental stress, may suggest that stress during the holiday period may not be fleeting but may instead consolidate into more enduring forms of strain with wider psychological consequences for parents and family relationships.

In terms of reciprocal dynamics, cognitive labour and subjective stress were found to share cross-lagged effects, with each variable predicting the other from one measurement point to the next. In addition, at the between-person level, a reciprocal association was also found between stress and cognitive labour, mirroring the within-person results. The reciprocal links between cognitive labour and stress at both levels indicate a self-reinforcing cycle, but with different implications. At the within-person level, the effects show that when parents experience a momentary spike in cognitive load, they feel more stressed shortly afterwards, where the increased stress then heightens cognitive labour in the next moment, creating a short-term spiral. At the between-person level, the same reciprocal pattern implies that parents who generally carry a higher mental load also tend to be more chronically stressed, and those who are chronically stressed tend to sustain higher overall cognitive labour, reflecting a longer-term, trait-like loop that may characterise differences between families during the holidays. Cognitive load, household labour and parental stress have previously been linked to stress^12,20^. However, due to the lack of suitable evidence of temporal dynamic patterns as provided by our ESM data, the interplay of these factors over time, how they may mutually influence each other and how this may result in gendered imbalances have thus far not been properly conceptualised or understood. Despite limitations by our design, the moment-to-moment temporal ordering of effects provides evidence of directional influence, and these reciprocal findings are consistent with, though not definitive proof of, a causal dynamic between the mental load and parental stress. This reciprocal relationship does carry implications for parenting intervention studies, as interventions targeted at parental stress^53,54^ may benefit from taking cognitive labour and its antecedents into account in the design and implementation of such interventions.

Furthermore, unidirectional effects were found for both subjective stress and cognitive labour predicting emotion suppression. Thus, when parents feel overloaded or stressed in a given moment, they are more likely to suppress their emotions shortly afterwards and rather display emotions that are not truly felt. These results point to a cascading pattern, where cognitive labour and stress mutually escalate and spill into emotion suppression, as parents may attempt to hide their levels of stress and cognitive overload from their children. The unidirectional nature of the results also indicates that emotion suppression may be a coping behaviour rather than a causal driver. These findings are in line with Gross’s process model of emotion regulation, where emotion suppression is conceptualised as a response-focused regulation strategy^8^. In contrast to the moment-to-moment data, in the between-person effects, emotion suppression was not predicted by either cognitive labour or stress. The absence of these effects at the between-person level indicates that parents who are generally more stressed or who carry a heavier mental load are not, on average, more likely to suppress their emotions than others. This pattern suggests that emotion suppression is a situational coping response to acute fluctuations in stress and cognitive load, rather than a stable individual tendency linked to parents’ overall stress or mental load profiles. In terms of sociodemographic variables, the results indicate that these stress and labour burdens over Christmas were largely consistent across family types, with no effects of income, single parenthood, or having a child with special needs.

### Mothers’ increased performance of cognitive labour without raised stress responses

Mothers reported performing more cognitive labour than fathers, even after controlling for earlier levels of cognitive labour. This finding is consistent with previous work suggesting that the invisible planning, organising, and monitoring involved in family life remains disproportionately undertaken by mothers despite broader changes in the division of domestic labour^55^. Such inequalities have been linked to persistent gender norms and expectations regarding responsibility for household organisation and childcare, which may contribute to the unequal distribution of cognitive labour within families^32^. In contrast, we observed no gender differences in stress or emotion suppression. This pattern suggests that the greater cognitive labour reported by mothers was not accompanied by corresponding differences in these psychological outcomes during the study period. Although emotional labour has been argued to be 'disproportionately associated with feminised work'^3^ (p. 18)—such as parenting—and mothers are often seen as responsible for managing the emotions of the family, we found no sex differences in emotion suppression. The mechanisms underlying this apparent discrepancy remain unclear. One possibility is that mothers and fathers differ in how they regulate or respond to the demands associated with cognitive labour, although the present data do not allow these mechanisms to be tested directly. Future research should therefore examine whether individual differences in emotion regulation, emotional resilience, or other psychological resources help explain why greater cognitive labour does not necessarily coincide with higher reported stress or emotion suppression in mothers.

### The role of social support: a relationship solution to a relationship problem?

Although previous literature has concluded that social support may be beneficial in lowering parental stress^21^, pre-survey social support did not predict either parental stress or cognitive labour at post-survey levels but did negatively predict emotion suppression. These findings suggest that the protective effects of social support are specific rather than global. Social support did not reduce parents’ overall stress or cognitive labour over the Christmas period, indicating that the mental load and stress cycle is relatively resistant to broader contextual resources—perhaps because social support can hardly reduce the required cognitive labour, but only aid in coping with the resulting stress, e.g. by providing opportunities for emotional regulation. Fittingly, parents who felt more supported reported significantly less emotion suppression at post-survey, suggesting that social support may ease the emotional burden of 'putting on a brave face'. This implies that social support helps parents cope emotionally, but does not fundamentally change the structural demands that generate stress and cognitive labour.

### Strengths and limitations

One of the study’s key strengths is the timing and frequency of data collection. The holiday period is a prime example for studying parental stress for multiple reasons: childcare duties are intensified during this period because of school holidays, and moreover, the season places heightened cultural expectations of emotional performance and seasonal activities on parents, all of which increases the required amounts of parenting labour to be performed. Moreover, it is rare to have data available continuously for over a month; the resulting dataset from daily measures over such a prolonged time is uniquely well suited for investigating the temporal dimension of parental stress and the gendered labour divide while also resulting in well-powered findings. Finally, studies on cognitive labour in the household often focus on mothers due to pre-existing findings that mothers bear more of the workload; while this is true, it also reinforces the idea that household labour, and specifically cognitive labour is something that by default concerns women, but not men. By contrast, our study included mothers and fathers.

At the same time, our study is in some ways limited by the fact that, while it included mothers and fathers, they were not couples and hence, the data are not dyadic. Dyadic data were not strictly required for the central questions of this study, but could have provided relevant additional information, in particular on the distribution of cognitive labour within couples and diverging perceptions thereof. It is also noteworthy that quantitative measurement of cognitive labour continues to be difficult; while the experience sampling design we selected mitigates some of the common issues (recall and related biases), it does not solve the issue that cognitive labour is in many ways inherently difficult to quantify. Thus, our measure is imperfect, and we run the risk of underestimating the extent and burden of parental cognitive labour. Furthermore, our sample consisted of British parents, overwhelmingly from families who celebrate Christmas, meaning the sample was culturally limited. In different cultural contexts, parenting norms and norms around motherhood may be different, resulting in different expectations and demands on emotion suppression and cognitive labour. Table 2 discusses the study’s limitations in detail.

**Table 2:** Assessment of limitations

Dimension	Assessment
Internal validity	The phenomenon was investigated using high-intensity longitudinal methods. To minimise the underestimation of cognitive labour that is commonly reported when attempting to assess the time spent on cognitive labour, which is difficult to estimate, we instead measure the number of everyday situations and activities during which participants were performing family-related cognitive labour throughout the day.
Statistical validity	The reliability of the measures used in this study was established as at least good, based on our dataset. The statistical power was sufficient. Our only control variables were socio-demographic or relating to the specific family model (e.g. whether parents were single). None of these variables can be affected by the measurements performed. However, stress could be affected by regular assessment via drawing the participants’ attention to the fact that they are stressed, thereby increasing the stress.
Generalizability to different methods	We only used self-report measures, but no measures of behaviour. Given the subjective nature of all constructs of interest in our study, this is largely unavoidable. We also relied on quantitative measures. Previous studies have found that the burden of cognitive household labour is described much more extensively in qualitative studies, as the phenomenon is hard to quantify for those affected.
Generalizability to field settings	The phenomenon was assessed in a field setting. ESM is a method known for its high ecological validity.
Generalizability to times and populations	Our findings are culturally and socio-economically specific. Firstly, centring the study on Christmas time and collecting data in the UK means the findings speak to a Western/European, at least culturally Christian, context. While the pressures of childcare during school holidays are likely to generalise, the specific cultural expectations of emotional performance and creation of Christmas magic may not transfer to other holidays celebrated in other population groups. Additionally, the parental norms and expectations that prompt parents to display certain emotions and hide others, as well as the extent to which family and parenting are considered 'women’s issues' and to which it is acceptable to express stress about parenting and to ask for social support, differ between cultural contexts. Finally, our sample was collected via Prolific and is thus particular in multiple ways; one crucial limitation was the limited range of socio-economic status. For those of lower socio-economic status who are facing financial pressures that may be exacerbated by holiday-related expenses, the extent and impact of parental stress and invisible labour may differ.
Theoretical limitations	Emotion suppression was conceptualised in the present study as the suppression of negative emotions and the display of positive emotions during interactions with children. This conceptualisation overlaps substantially with the surface acting dimension of emotional labour, and previous literature has suggested that these constructs share considerable conceptual commonality^9^. In line with this overlap, emotion suppression in the present study was measured using adapted items from the surface acting subscale of the Emotional Labour Scale^40^. However, there remains ongoing conceptual uncertainty within the literature regarding the extent to which surface acting should be understood as a form of emotion regulation, emotional labour, or both^9^. As such, the present findings should be interpreted specifically as reflecting suppressive emotional regulation in parenting contexts and not as the full broader construct of emotional labour. Furthermore, because the study focused specifically on suppressing and masking emotions, it does not capture other emotion regulation strategies, such as cognitive reappraisal or deep acting, which involve attempts to genuinely modify one’s internal emotional experience rather than merely regulate its outward display^8,10^. These alternative strategies may show different temporal dynamics and associations with stress and cognitive labour. Future research should therefore continue clarifying the conceptual and empirical distinctions between emotional labour, emotion regulation, and emotion suppression within parenting contexts.

## Conclusion

This study provides insight into how invisible parental labour unfolds during the Christmas season, a period characterised by heightened cultural expectations and intensified childcare demands. By using experience sampling methods across 35 days, we captured momentary fluctuations in cognitive labour, emotion suppression, and parental stress, revealing that these processes are both highly stable and deeply intertwined. Cognitive labour and stress formed a reciprocal, self-reinforcing loop at both the within- and between-person levels, highlighting how mental load and stress accumulate and sustain one another in everyday parenting. In contrast, emotion suppression functioned as a situational coping response, emerging in reaction to spikes in cognitive labour and stress but not predicting them in return. At the same time, we found limited gender differences beyond cognitive labour. Although mothers reported assuming a greater share of cognitive labour than fathers, they did not report higher stress or emotion suppression. This finding raises important questions about the mechanisms through which unequal cognitive labour translates into psychological outcomes. Nevertheless, the absence of immediate differences in stress should not be interpreted as evidence that persistent inequalities in cognitive labour are benign or without longer-term consequences. Social support showed a targeted, rather than global, protective effect by reducing emotion suppression but not altering broader stress–labour dynamics. Together, these findings highlight the detrimental imbalance in cognitive labour performed by women in parenting partnerships and show that these effects cannot be mitigated by external social support. This indicates that to reduce the mental load burdening mothers, it is not enough to support them within the existing system: rather, the cultural norms around parenting and the gendered stickiness of cognitive labour must be addressed, and parenting couples’ dynamics directly targeted to improve women’s mental burden in the household.

## Supplementary information

Transparent Peer Review File

Supplementary Information

## Data Availability

All data used in this study is available at: https://osf.io/ad647.

## Code availability

All code used in this study is available at: https://osf.io/ad647.

## References

[1] Hays, S. *The Cultural Contradictions of Motherhood* (Yale University Press, 1998).

[2] Parker, K. & Wang, W. *Modern Parenthood.* Pew Research Center’s Social & Demographic Trends Project, Vol. 14, 1–66 (Pew Research Center, 2013).

[3] Dean, L., Churchill, B. & Ruppanner, L. The mental load: building a deeper theoretical understanding of how cognitive and emotional labor overload women and mothers. *Community Work Fam.* **25**, 13–29 10.1080/13668803.2021.2002813 (2022).

[4] Copeland, D. & Harbaugh, B. L. Differences in parenting stress between married and single first time mothers at six to eight weeks after birth. *Issues Compr. Pediatr. Nurs.* **28**, 139–152 10.1080/01460860500227556 (2005). 16251160

[5] Martins, P. C., Matos, C. D. & Sani, A. I. Parental stress and risk of child abuse: the role of socioeconomic status. *Child. Youth Serv. Rev.* **148**, 106879 10.1016/j.childyouth.2023.106879 (2023).

[6] Oyarzún-Farías, M. D. L. A., Cova, F. & Bustos Navarrete, C. Parental stress and satisfaction in parents with pre-school and school age children. *Front. Psychol.* **12**, 683117 10.3389/fpsyg.2021.683117 (2021). PMC8195280 34122279

[7] Lin, G. X. et al. Parenting with a smile: display rules, regulatory effort, and parental burnout. *J. Soc. Personal. Relatsh.* **38**, 2701–2721 10.1177/02654075211019124 (2021).

[8] Gross, J. J. & John, O. P. Individual differences in two emotion regulation processes: implications for affect, relationships, and well-being. *J. Personal. Soc. Psychol.* **85**, 348 10.1037/0022-3514.85.2.348 (2003). 12916575

[9] Grandey, A. A. & Melloy, R. C. The state of the heart: emotional labor as emotion regulation reviewed and revised. *J. Occup. Health Psychol.* **22**, 407–422 10.1037/ocp0000067 (2017). 28150996

[10] Hochschild, A. R. The Managed Heart: Commercialization of Human Feeling. (University of California Press, 1983).

[11] Le, B. M. & Impett, E. A. The costs of suppressing negative emotions and amplifying positive emotions during parental caregiving. *Personal. Soc. Psychol. Bull.* **42**, 323–336 10.1177/0146167216629122 (2016). 26865288

[12] Ciciolla, L. & Luthar, S. S. Invisible household labor and ramifications for adjustment: mothers as captains of households. *Sex. Roles* **81**, 467–486 10.1007/s11199-018-1001-x (2019). PMC8223758 34177072

[13] Daly, K. Time, gender, and the negotiation of family schedules. *Symb. Interact.* **25**, 323–342 10.1525/si.2002.25.3.323 (2002).

[14] Wayne, J. H., Mills, M. J., Wang, Y. R., Matthews, R. A. & Whitman, M. V. Who’s remembering to buy the eggs? The meaning, measurement, and implications of invisible family load. *J. Bus. Psychol.* **38**, 1159–1184 10.1007/s10869-023-09887-7 (2023). PMC10228438 37359080

[15] Daminger, A. The cognitive dimension of household labor. *Am. Sociol. Rev.* **84**, 609–633 10.1177/0003122419859007 (2019).

[16] Barigozzi, F. et al. Beyond time: unveiling the invisible burden of mental load. Preprint at 10.48550/arXiv.2505.11426 (2025).

[17] Schrodt, P. & O’Mara, C. The development and validation of the emotion labor in families scale: associations with emotion regulation, feeling caught, and relational satisfaction in parent-child relationships. *Commun. Q.* **67**, 383–404 10.1080/01463373.2019.1596143 (2019).

[18] Waters, S. F., Karnilowicz, H. R., West, T. V. & Mendes, W. B. Keep it to yourself? Parent emotion suppression influences physiological linkage and interaction behavior. *J. Fam. Psychol.* **34**, 784 10.1037/fam0000664 (2020). 32324017

[19] Zimmer-Gembeck, M. J., Rudolph, J., Kerin, J. & Bohadana-Brown, G. Parent emotional regulation: a meta-analytic review of its association with parenting and child adjustment. *Int. J. Behav. Dev.* **46**, 63–82 10.1177/01650254211051086 (2022).

[20] Aviv, E. et al. Cognitive household labor: gender disparities and consequences for maternal mental health and wellbeing. *Arch. Women’s Ment. Health* **28**, 5–14 10.1007/s00737-024-01490-w (2025). PMC11761833 38951218

[21] Park, G. A. & Lee, O. N. The moderating effect of social support on parental stress and depression in mothers of children with disabilities. *Occup. Ther. Int.* **2022**, 5162954 10.1155/2022/5162954 (2022). PMC8938151 35359427

[22] Gross, J. J. The emerging field of emotion regulation: an integrative review. *Rev. Gen. Psychol.* **2**, 271–299 10.1037/1089-2680.2.3.271 (1998).

[23] Lin, G. X. & Szczygieł, D. Emotional labor in parenting. in (eds Roskam, I., Gross, J. J. & Mikolajczak, M.) *Emotion Regulation and Parenting,* 244–263 (Cambridge University Press, 2024).

[24] Karnilowicz, H. R., Waters, S. F. & Mendes, W. B. Not in front of the kids: effects of parental suppression on socialization behaviors during cooperative parent–child interactions. *Emotion* **19**, 1183–1191 10.1037/emo0000527 (2019). PMC6764913 30475030

[25] Lin, G. X. et al. Parenting culture(s): ideal-parent beliefs across 37 countries. *J. Cross Cult. Psychol.* **54**, 4–24 10.1177/00220221221123043 (2023).

[26] Gross, J. J. & Levenson, R. W. Hiding feelings: the acute effects of inhibiting negative and positive emotion. *J. Abnorm. Psychol.* **106**, 95–103 10.1037/0021-843X.106.1.95 (1997). 9103721

[27] Richards, J. M. & Gross, J. J. Emotion regulation and memory: the cognitive costs of keeping one’s cool. *J. Personal. Soc. Psychol.* **79**, 410–424 10.1037/0022-3514.79.3.410 (2000). 10981843

[28] Goldin, P. R., McRae, K., Ramel, W. & Gross, J. J. The neural bases of emotion regulation: reappraisal and suppression of negative emotion. *Biol. Psychiatry* **63**, 577–586 10.1016/j.biopsych.2007.05.031 (2008). PMC2483789 17888411

[29] Petts, R. J. & Carlson, D. L. Managing a household during a pandemic: cognitive labor and parents’ psychological well-being. *Soc. Ment. Health* **13**, 187–207 10.1177/21568693231169521 (2023).

[30] Robertson, L. G., Anderson, T. L., Hall, M. E. L. & Kim, C. L. Mothers and mental labor: a phenomenological focus group study of family-related thinking work. *Psychol. Women Q.* **43**, 184–200 10.1177/0361684319825581 (2019).

[31] Reding, C. & Smith, L. I have to think for four people”: Belgian mothers’ mental labour during the coronavirus lockdowns. *J. Undergrad. Ethnogr.* **12**, 17–32 10.15273/jue.v12i1.11311 (2022).

[32] Reich-Stiebert, N., Froehlich, L. & Voltmer, J. B. Gendered mental labor: a systematic literature review on the cognitive dimension of unpaid work within the household and childcare. *Sex. Roles* **88**, 475–494 10.1007/s11199-023-01362-0 (2023). PMC10148620 37283733

[33] Miller, D. Christmas: an anthropological lens. *HAU J. Ethnogr. Theory* **7**, 409–442 10.14318/hau7.3.027 (2017).

[34] Schneider, E., Liwinski, T., Imfeld, L., Lang, U. E. & Brühl, A. B. Who is afraid of Christmas? The effect of Christmas and Easter holidays on psychiatric hospitalizations and emergencies—systematic review and single center experience from 2012 to 2021. *Front. Psychiatry* **13**, 1049935 10.3389/fpsyt.2022.1049935 (2023). PMC9874097 36713912

[35] Syrek, C. J., Weigelt, O., Kühnel, J. & de Bloom, J. All I want for Christmas is recovery–changes in employee affective well-being before and after vacation. *Work Stress* **32**, 313–333 10.1080/02678373.2018.1427816 (2018).

[36] Hairon, N. How Christmas festivities and pressures can damage health and well-being. *Nursing* **104**, 33–34 (2008). 19165982

[37] English, C. & Brown, G. My mum is on strike! Social reproduction and the (emotional) labor of ‘mothering work’ in neoliberal Britain. *Gend. Work Organ.* **30**, 1941–1959 10.1111/gwao.13027 (2023).

[38] Teuber, Z. et al. Higher momentary parental burnout predicts lower subsequent emotional expression in parents during the festive season. *Commun. Psychol.* **3**, 167 10.1038/s44271-025-00346-y (2025). PMC12644506 41286517

[39] Office for National Statistics. *Average Household Income* (UK-Office for National Statistics, 2024).

[40] Yang, C., Chen, Y. & Zhao, X. Emotional labor: scale development and validation in the Chinese context. *Front. Psychol.* **10**, 2095 10.3389/fpsyg.2019.02095 (2019). PMC6759872 31620048

[41] Wong, S. M. Y. et al. Measuring subjective stress among young people in Hong Kong: validation and predictive utility of the single-item subjective level of stress (SLS-1) in epidemiological and longitudinal community samples. *Epidemiol. Psychiatr. Sci.* **30**, e61 10.1017/S2045796021000445 (2021).

[42] Kliem, S. et al. A brief form of the Perceived Social Support Questionnaire (F-SozU) was developed, validated, and standardized. *J. Clin. Epidemiol.* **68**, 551–562 10.1016/j.jclinepi.2014.11.003 (2015). 25499982

[43] Nezlek, J. B. A practical guide to understanding reliability in studies of within-person variability. *J. Res. Personal.* **69**, 149–155 10.1016/j.jrp.2016.06.020 (2017).

[44] Schuurman, N. K. & Hamaker, E. L. Measurement error and person-specific reliability in multilevel autoregressive modeling. *Psychol. Methods* **24**, 70–91 10.1037/met0000188 (2019). 30188157

[45] Hamaker, E. L., Asparouhov, T., Brose, A., Schmiedek, F. & Muthén, B. At the frontiers of modeling intensive longitudinal data: dynamic structural equation models for the affective measurements from the COGITO study. *Multivar. Behav. Res.* **53**, 820–841 10.1080/00273171.2018.1446819 (2018). 29624092

[46] McNeish, D. & Hamaker, E. L. A primer on two-level dynamic structural equation models for intensive longitudinal data in Mplus. *Psychol. Methods* **25**, 610–635 10.1037/met0000250 (2020). 31855015

[47] Muthén, L. K. & Muthén, B. O. *Mplus 9* (Muthén & Muthén, 2025).

[48] Gelman, A. & Rubin, D. B. Inference from iterative simulation using multiple sequences. *Stat. Sci.* **7**, 457–472 10.1214/ss/1177011136 (1992).

[49] Maroufy, H. E., Hibbah, E. H., Zyad, A. & Taib, Z. Bayesian estimation of multivariate autoregressive hidden Markov model with application to breast cancer biomarker modeling. in (ed. Tejedor, J. P.) *Bayesian Inference*, 147–166 10.5772/intechopen.70053 (InTek, 2017).

[50] Roskam, I., Raes, M. E. & Mikolajczak, M. Exhausted parents: development and preliminary validation of the parental burnout inventory. *Front. Psychol.* **8**, 236360 10.3389/fpsyg.2017.00163 (2017). PMC5298986 28232811

[51] Johnson, M. S., Skjerdingstad, N., Ebrahimi, O. V., Hoffart, A. & Johnson, S. U. Parenting in a pandemic: parental stress, anxiety and depression among parents during the government-initiated physical distancing measures following the first wave of COVID-19. *Stress Health* **38**, 637–652 10.1002/smi.3120 (2022). 34902219

[52] Senn, M., Stadelmann, C., Forster, F., Nussbeck, F. W. & Bodenmann, G. Parental stress mediates the effects of parental risk factors on dysfunctional parenting in first-time parents: a dyadic longitudinal study. *J. Soc. Personal. Relatsh.* **40**, 4335–4358 10.1177/02654075231165340 (2023). PMC10695745 38058532

[53] Potharst, E. S. et al. A randomized control trial evaluating an online mindful parenting training for mothers with elevated parental stress. *Front. Psychol.* **10**, 1550 10.3389/fpsyg.2019.01550 (2019). PMC6650592 31379646

[54] Urbanowicz, A. M. et al. Cognitive behavioral stress management for parents: Prevention and reduction of parental burnout. *Int. J. Clin. Health Psychol.* **23**, 100365 10.1016/j.ijchp.2023.100365 (2023). PMC9999161 36908695

[55] Weeks, A., Kowalewska, H. & Ruppanner, L. Take a load off? Not for mothers: gender, cognitive labor, and the limits of time and money. *Socius Sociol. Res. Dyn. World* **11** 10.1177/23780231251384527 (2025).

