# Supplementary material for: Parenting stress spirals and a gendered labour division over the Christmas holidays
Source: Commun Psychol. 2026 Oct 24;4:149. doi: 10.1038/s44271-026-00535-3 (PMC13695652; doi:10.1038/s44271-026-00535-3)
Supplement: Supplementary file 1 [file 44271_2026_535_MOESM1_ESM.pdf]

nature portfolio

Peer Review File

Parenting stress spirals and a gendered labour division over the Christmas holidays

Corresponding Author: Dr Ziwen Teuber

This file contains all reviewer reports in order by version, followed by all author rebuttals in order by version.

Version 0:

Reviewer comments:

Reviewer #1

(Remarks to the Author)
This manuscript uses an EMA approach during the Christmas holiday season to examine parents' stress, and cognitive and emotional demands. The authors find that parents stress and cognitive demands were reciprocally related (more stress more demands and vice versa) and emotional demands, what the authors call "surface acting," was predicted by stress and cognitive demands. Mothers reported more cognitive demands (operationalized as planning and organizing around the holidays) than fathers but stress and emotional demands did not differ between mothers and fathers.

I enjoyed reading this paper. It asked an interesting and novel question and used a time period that is typically fraught with lots of demands, cultural expectations, and strife. The EMA approach was well conceived to ask these questions, and the results were clearly described. I wish the authors had collected dyadic data here to test some of the assumptions regarding perceptions of labour versus actual labour within a couple, but maybe that could be a follow-up study for them. I will mention that it was not clear to me that the authors did not collect dyadic data until the limitation section. I suggest being a bit clearer on that issue in the method section. I found myself going back to the method section when reading the results to figure out if the husband/wives were couples or not.

I included a few observations I had while reading the manuscript.

1. Surface acting or suppression? Every area and sub-area uses different language and labels, which is not great for a coherent discipline, but certainly understandable. This comment is really just food for thought for the authors, but when I read the operationalization of "emotional labour" I wondered if there is a better psychological label given the item collected. In the EMA data the authors collected a single item "I am putting on an act to deal with my children in an appropriate way." The authors then call this "surface acting." That does seem to be a reasonable label but there is a psychological construct that is well studied and seems to fit better and that is emotional suppression. I wondered if looking at the broader literature on emotion suppression might make sense here.

2. Leveraging the arc of the holiday season. As noted, the holiday season is an ideal context to examine these processes. Did the authors consider leveraging the arc of the season where one could examine how stress/demands increase as Christmas approaches but then once the day passes there are not as many demands? Maybe the inertia observed is for pre-holiday stress/demands and then there is a shift afterwards? Like a regression discontinuity design with Christmas Day as the critical event/treatment.

3. Interpretation of findings. I think it's clear that I like this paper and am supportive of the work, so I include this last point in the spirit of addressing a reaction I had that other readers may have as well. Specifically, I was unsettled by some of the authors' interpretations of their findings in which they over-stepped the appropriate line of interpreting their data and engaged in inferring psychological states for which they have no evidence. The authors found that mothers reported more cognitive labour than fathers, but they observed no gender differences in reported stress or "emotional labour." Instead of simply accepting these null findings the authors engage in quite a bit of tortured speculation that women were either not reporting what they really feel because of cultural expectations or suggesting that women are more resilient and that's why these other outcomes did not show gendered differences. I found these arguments both infantilizing of women, but also, more seriously, bad scholarship. These explanations came off as politically or personally motivated rather than researchers reporting what they found in an unbiased way. If the authors really believe that women would hold back on reporting their

authentic stress responses, then why did they collect self-report stress responses? There are other strategies researchers use if they are concerned about biased reporting in stress such as physiological responses, partner reports, behavior, or implicit outcomes. If the authors had observed a gendered difference in stress and/or emotional labour, I am certain they would have been happy to present those findings without these explanations. At a time when science and research is under attack, we have a responsibility to report studies faithfully and not project our beliefs on how the data should have turned out. Maybe the authors are wrong about their beliefs that women should report more stress and "emotional labour" than men, they certainly did not find evidence of it. Trying to interpret the null findings with these various speculations takes the work out of the empirical realm and into wishful thinking.

Reviewer #2

(Remarks to the Author)

This manuscript addresses parental emotional and cognitive labor, and their relatopnship to parental stress, using an intensive longitudinal design. It offers novel conceptual framing and robust empirical work that advances understanding of gendered experiences of parenting-related stress and regulation.
Overall, and despite the very short measures (which are typical of intensive longitudinal designs, though), this is a strong and original manuscript with important methodological, conceptual and practical contributions.

Major strengths
- The introduction, and particularly the first paragraph, is original and engaging, providing an appealing entry to the topic.
- The rest of the introduction is also excellent, with references to all the most recent and relevant studies. I could not think of any reference that was missed.
- The distinction between emotional labor and cognitive labor is well made and important; treating these as separate constructs strengthens conceptual clarity and treating them both in the same study increases our understanding of the invisible contributors to parental stress.
- Methodologically, the use of an intensive longitudinal design is a major asset of this study. Such designs are underused in this literature and provide valuable temporal resolution for understanding parental stress processes. Congratulations to the authors.
- The detailed table (Table 2) presenting an in-depth assessment of measurement boundaries/limits is excellent and exemplary. It was the very first time that I saw that in my career and I loved it. it represents a desirable and useful model for all scientific papers.
- The discussion is absolutely excellent. I anticipate a potential resistance to findings (e.g., from male reviewers) and I hope that this will not prevent the publication of this paper.

Concerns and recommendations
1. In the first paragraph of the introduction or shortly thereafter, the authors should more clearly describe the concrete obligations that occur during this period—particularly multiple family gatherings and extensive gift-giving. Such obligations can influence both the frequency and type of cognitive and emotional labor parents perform (e.g., finding appropriate gifts for many people, concealing from children that one does not want to attend certain family events). A few more concrete details about what the holiday period entails would help readers from other cultural or religious backgrounds appreciate the emotional, cognitive, and practical burdens placed on parents (especially mothers) during this time.

2. Emotional labor measurement: Emotional labor is represented only by surface acting in the current operationalization. Classic emotional labor theory distinguishes surface acting and deep acting as qualitatively different regulation strategies. The authors tried to justify why the only selected surface acting,; but I was not convinced. I recommend one of the options below:
- Preferably, run all analyses twice, one with surface acting, one with deep acting. If word count is an issue, results pertaining to deep acting could be put in supplementary online material and convergence/divergence between results could be discussed in the General Discussion.
- Create a combined emotional labour score (and justify its construction) and run the models with this combined score. Models with SA and DA alone would be presented in Online Supplemental Material, and convergence/divergence between results could be discussed in the General Discussion.

Version 1:

Reviewer comments:

Reviewer #1

(Remarks to the Author)
Thank you for resubmitting a responsive letter and revised manuscript. I found the emotion regulation framing well aligned with the data collected and I hope this shift in framing is useful in engaging the large body of emotion regulation researchers. I also appreciated the interpretations in the discussion. Excellent revision.

Reviewer #2

(Remarks to the Author)
Thank you for revising the paper and taking our comments into account. I am satisfied with the revised version.

Open Access This Peer Review File is licensed under a Creative Commons Attribution 4.0 International License, which permits use, sharing, adaptation, distribution and reproduction in any medium or format, as long as you give appropriate credit to the original author(s) and the source, provide a link to the Creative Commons license, and indicate if changes were made.
In cases where reviewers are anonymous, credit should be given to 'Anonymous Referee' and the source.
The images or other third party material in this Peer Review File are included in the article’s Creative Commons license, unless indicated otherwise in a credit line to the material. If material is not included in the article’s Creative Commons license and your intended use is not permitted by statutory regulation or exceeds the permitted use, you will need to obtain permission directly from the copyright holder.
To view a copy of this license, visit https://creativecommons.org/licenses/by/4.0/

Dear Prof. Hannah Hao and Reviewers

Thank you for the positive evaluation of our manuscript and the helpful comments and suggestions for improvement. We have addressed all of the comments individually below and have indicated the changes in the manuscript in yellow highlighter.

In general, the following major changes were made to the manuscript:

- Following the helpful comments of both reviewers, we have re-engaged with the literature on emotion suppression and emotion regulation and have reframed our 'emotion labour' variable as 'emotion suppression'. We believe this label more clearly describe its measurement. In addition, we also provided an overview of the conceptual overlap of emotion suppression as viewed through an emotion regulation lens and surface acting as viewed through an emotional labour lens in order to make it clear to the reader that considerable conceptual overlap exists in the literature. This has led to major changes in the introduction, literature review, discussion, and limitation sections.
- We have made the non-dyadic nature of our data clearer in the methods section.
- We have added examples of the expectations of the Christmas season in order to make the study more accessible for readers outside of a Western cultural context.
- The discussion section has been adjusted to exclude speculations about the impact of self-report assessment on the validity of stress measures used in this study; at the same time, we have revised the discussion to provide a more cautious interpretation of the observed gender effects.

Reviewer #1:

This manuscript uses an EMA approach during the Christmas holiday season to examine parents' stress, and cognitive and emotional demands. The authors find that parents stress and cognitive demands were reciprocally related (more stressà more demands and vice versa) and emotional demands, what the authors call "surface acting," was predicted by stress and cognitive demands. Mothers reported more cognitive demands (operationalized as planning and organizing around the holidays) than fathers but stress and emotional demands did not differ between mothers and fathers.

I enjoyed reading this paper. It asked an interesting and novel question and used a time period that is typically fraught with lots of demands, cultural expectations, and strife. The EMA approach was well conceived to ask these questions, and the results were clearly described. I wish the authors had collected dyadic data here to test some of the assumptions regarding perceptions of labour versus actual labour within a couple, but maybe that could be a follow-up study for them. I will mention that it was not clear to me that the authors did not collect dyadic data until the limitation section. I suggest being a bit clearer on that issue in the method section. I found myself going back to the method section when reading the results to figure out if the husband/wives were couples or not.

Thank you very much for this evaluation of our manuscript. We fully agree that the non-dyadic nature of the data ought to be stated explicitly. We have therefore added this statement in the Methods | Participants section: "Participants were recruited and analysed as individual parents rather than parent dyads; thus, mothers and fathers in the sample were not partners within the same household."

I included a few observations I had while reading the manuscript.

1. Surface acting or suppression? Every area and sub-area uses different language and labels, which is not great for a coherent discipline, but certainly understandable. This comment is really just food for thought for the authors, but when I read the operationalization of "emotional labour" I wondered if there is a better psychological label given the item collected. In the EMA data the authors collected a single item "I am putting on an act to deal with my children in an appropriate way." The authors then call this "surface acting." That does seem to be a reasonable label but there is a psychological construct that is well studied and seems to fit better and that is emotional suppression. I wondered if looking at the broader literature on emotion suppression might make sense here.

We want to sincerely thank the reviewer for this comment. We agree that the terminology surrounding emotional labour, surface acting, and emotion suppression is often overlapping and inconsistently used across different literatures/disciplines. Following your suggestion, we

substantially revised the manuscript to reframe the focal construct as emotion suppression rather than emotional labour/surface acting.

More specifically, we incorporated the broader emotion regulation literature into the theoretical framing and now conceptualise the construct in line with Gross's process model of emotion regulation (Gross, 1998; Gross & John, 2003). We define emotion suppression as a response-focused emotion regulation strategy involving the inhibition or masking of outward emotional expression. We then explain that this construct overlaps conceptually with the surface acting dimension of emotional labour, as both involve suppressing genuinely felt emotions and displaying socially expected emotions instead.

To reflect this reframing, we revised the Introduction and theoretical sections to distinguish more clearly between emotional labour, surface acting, and emotion suppression. We also changed the terminology throughout the manuscript from "emotional labour" and "surface acting" to "emotion suppression" where appropriate, including the abstract, research questions, methods, results, discussion, figures, and tables. In addition, we clarified in the limitations section that the construct was operationalised using adapted items from the surface acting subscale of the Emotional Labour Scale, and that there remains some conceptual overlap and ambiguity in the literature regarding the extent to which surface acting should be understood as emotional labour, emotion regulation, or both.

We believe this reframing substantially improved the conceptual clarity of the manuscript and better aligns the operationalisation with the broader psychological literature on expressive suppression and emotion regulation.

In doing so, we have also added the following references:

- Grandey, A. A. & Melloy, R. C. (2017). The State of the Heart: Emotional Labor as Emotion Regulation Reviewed and Revised. Journal of Occupational Health Psychology, 22 (3), 407-422. doi: 10.1037/ocp0000067.
- Gross, J. J. (1998). The emerging field of emotion regulation: An integrative review. Review of General Psychology, 2, 271–299. http://dx.doi .org/10.1037/1089-2680.2.3.271
- Gross, J. J., & John, O. P. (2003). Individual differences in two emotion regulation processes: implications for affect, relationships, and well-being. Journal of personality and social psychology, 85(2), 348. https://psycnet.apa.org/doi/10.1037/0022-3514.85.2.348
- Karnilowicz, H. R., Waters, S. F., & Mendes, W. B. (2019). Not in front of the kids: Effects of parental suppression on socialization behaviors during cooperative parent–child interactions. Emotion, 19(7), 1183–1191. https://doi.org/10.1037/emo0000527
- Waters, S. F., Karnilowicz, H. R., West, T. V., & Mendes, W. B. (2020). Keep it to yourself? Parent emotion suppression influences physiological linkage and interaction

behavior. Journal of Family Psychology, 34(7), 784. https://psycnet.apa.org/doi/10.1037/fam0000664

- Zimmer-Gembeck, M. J., Rudolph, J., Kerin, J., & Bohadana-Brown, G. (2022). Parent emotional regulation: A meta-analytic review of its association with parenting and child adjustment. International Journal of Behavioral Development, 46(1), 63-82. https://doi.org/10.1177/01650254211051086

2. Leveraging the arc of the holiday season. As noted, the holiday season is an ideal context to examine these processes. Did the authors consider leveraging the arc of the season where one could examine how stress/demands increase as Christmas approaches but then once the day passes there are not as many demands? Maybe the inertia observed is for pre-holiday stress/demands and then there is a shift afterwards? Like a regression discontinuity design with Christmas Day as the critical event/treatment.

Thank you for this inspiring comment and questions. Initially, we ran two DSEMs: The DSEM reported in the current manuscript. Then, to ensure the reported within-person dynamics were not confounded by a temporal trend, we re-estimated the model as a residual DSEM including a linear effect of time on all constructs at the within-person level; the time effect was negligible and the autoregressive and cross-lagged coefficients were virtually identical to those in the reported model. We found that the effect of Time was minimal and the coefficients were almost indifferent. We decided to keep the first model, which was much less complex and easier to interpret.

3. Interpretation of findings. I think it's clear that I like this paper and am supportive of the work, so I include this last point in the spirit of addressing a reaction I had that other readers may have as well. Specifically, I was unsettled by some of the authors' interpretations of their findings in which they over-stepped the appropriate line of interpreting their data and engaged in inferring psychological states for which they have no evidence. The authors found that mothers reported more cognitive labour than fathers, but they observed no gender differences in reported stress or "emotional labour." Instead of simply accepting these null findings the authors engage in quite a bit of tortured speculation that women were either not reporting what they really feel because of cultural expectations or suggesting that women are more resilient and that's why these other outcomes did not show gendered differences. I found these arguments both infantilizing of women, but also, more seriously, bad scholarship. These explanations came off as politically or personally motivated rather than researchers reporting what they found in an unbiased way. If the authors really believe that women would hold back on reporting their authentic stress responses, then why did they collect self-report stress responses? There are other strategies researchers use if they are concerned about biased reporting in stress such as physiological responses, partner reports, behavior, or implicit outcomes. If the authors had

observed a gendered difference in stress and/or emotional labour, I am certain they would have been happy to present those findings without these explanations. At a time when science and research is under attack, we have a responsibility to report studies faithfully and not project our beliefs on how the data should have turned out. Maybe the authors are wrong about their beliefs that women should report more stress and "emotional labour" than men, they certainly did not find evidence of it. Trying to interpret the null findings with these various speculations takes the work out of the empirical realm and into wishful thinking.

Thank you for this thoughtful and constructive comment. We agree that the discussion should remain closely grounded in the empirical findings and appreciate the reviewer's concerns regarding the extent of our interpretation. While we believe that considering potential explanations for both significant and meaningful null findings is an important function of the discussion section, we also recognise that some of our interpretations extended beyond what could be directly supported by the present data.

In response, we have substantially revised this section. We have removed the discussion concerning potential limitations of the self-report measures, as we agree that this was more speculative than warranted by the available evidence. We have also revised the discussion of the observed gender differences in cognitive labour alongside the absence of corresponding gender differences in stress to provide a more cautious and appropriately qualified interpretation. The revised text makes clearer that these explanations are offered as possible interpretations rather than conclusions supported directly by the data.

Reviewer #2

This manuscript addresses parental emotional and cognitive labor, and their relationship to parental stress, using an intensive longitudinal design. It offers novel conceptual framing and robust empirical work that advances understanding of gendered experiences of parenting-related stress and regulation.
Overall, and despite the very short measures (which are typical of intensive longitudinal designs, though), this is a strong and original manuscript with important methodological, conceptual and practical contributions.

Major strengths
- The introduction, and particularly the first paragraph, is original and engaging, providing an appealing entry to the topic.
- The rest of the introduction is also excellent, with references to all the most recent and relevant studies. I could not think of any reference that was missed.
- The distinction between emotional labor and cognitive labor is well made and important; treating these as separate constructs strengthens conceptual clarity and treating them both in the same study increases our understanding of the invisible contributors to parental stress.
- Methodologically, the use of an intensive longitudinal design is a major asset of this study. Such designs are underused in this literature and provide valuable temporal resolution for understanding parental stress processes. Congratulations to the authors.
- The detailed table (Table 2) presenting an in-depth assessment of measurement boundaries/limits is excellent and exemplary. It was the very first time that I saw that in my career and I loved it. it represents a desirable and useful model for all scientific papers.
- The discussion is absolutely excellent. I anticipate a potential resistance to findings (e.g., from male reviewers) and I hope that this will not prevent the publication of this paper.

Thank you very much for the positive evaluation of our manuscript and the helpful suggestions to improve, it is appreciated.

Concerns and recommendations
1. In the first paragraph of the introduction or shortly thereafter, the authors should more clearly describe the concrete obligations that occur during this period—particularly multiple family gatherings and extensive gift-giving. Such obligations can influence both the frequency and type of cognitive and emotional labor parents perform (e.g., finding appropriate gifts for many people, concealing from children that one does not want to attend certain family events). A few more concrete details about what the holiday period entails would help readers from other cultural or religious backgrounds appreciate the emotional, cognitive, and practical burdens placed on parents (especially mothers) during this time.

Thank you for this comment. We have expanded the first paragraph to give more concrete examples of the expectations surrounding Christmas in order to give more context to readers of other cultural or religious backgrounds: "During this period, parents are often expected to organise multiple family gatherings, coordinate travel plans to grandparents or other extended family, purchase and wrap gifts, prepare traditional meals, maintain family traditions such as decorating the Christmas tree, and manage children's expectations surrounding the holiday season. These obligations frequently require substantial cognitive planning and emotional regulation, such as concealing stress, frustration, or interpersonal tensions while maintaining a festive and positive atmosphere for children and extended family members."

2. Emotional labor measurement: Emotional labor is represented only by surface acting in the current operationalization. Classic emotional labor theory distinguishes surface acting and deep acting as qualitatively different regulation strategies. The authors tried to justify why the only selected surface acting,; but I was not convinced. I recommend one of the options below:
- Preferably, run all analyses twice, one with surface acting, one with deep acting. If word count is an issue, results pertaining to deep acting could be put in supplementary online material and convergence/divergence between results could be discussed in the General Discussion.
- Create a combined emotional labour score (and justify its construction) and run the models with this combined score. Models with SA and DA alone would be presented in Online Supplemental Material, and convergence/divergence between results could be discussed in the General Discussion.

Thank you for this thoughtful comment. We agree that classical conceptualisations of emotional labour distinguish between surface acting and deep acting as theoretically distinct regulatory processes. However, following the reviewer feedback and further engagement with the broader emotion regulation literature, we reconsidered the conceptual framing of the manuscript and substantially revised the paper accordingly.

Rather than positioning the focal construct as emotional labour broadly defined, we reframed the study specifically around emotion suppression. In the revised manuscript, we now conceptualise the measured construct as suppressing negative emotions while displaying socially expected positive emotions during interactions with children. We further situate this construct within Gross's process model of emotion regulation as a form of expressive suppression, while also explaining its conceptual overlap with the surface acting component of emotional labour.

As part of this reframing, we no longer interpret the construct as representing the broader multidimensional emotional labour framework encompassing both surface acting and deep acting. Instead, the study now explicitly focuses on suppressive emotional regulation in parenting contexts.

To improve conceptual transparency, we also added a dedicated limitations section acknowledging that the construct was measured using adapted items from the surface acting

subscale of the Emotional Labour Scale and that there remains ongoing conceptual overlap and ambiguity in the literature regarding the relationship between surface acting, emotional labour, and expressive suppression/emotion regulation. We believe that this reframing better aligns the operationalisation, theoretical framing, and interpretation of the findings throughout the manuscript.

Dear Prof. Hannah Hao and Reviewers

Thank you for the positive evaluation of our revised manuscript. We have now updated the manuscript based on the editorial comments, changed the reference style to the Nature referencing style, saved figure files separately with figure captions at the end of the document, and unblinded the ethical review panel.
