# Supplementary material for: Parenting stress spirals and a gendered labour division over the Christmas holidays
Source: Commun Psychol. 2026 Oct 24;4:149. doi: 10.1038/s44271-026-00535-3 (PMC13695652; doi:10.1038/s44271-026-00535-3)
Supplement: Supplementary file 2 [file 44271_2026_535_MOESM2_ESM.docx]

**Parenting stress spirals and a gendered labour division over the Christmas holidays**

**Supplement 1: Details on Procedure and Participants**

As reported in (Teuber et al., 2025):

The study was conducted from November 2023 to January 2024 and comprised a baseline survey, a 35-day experience sampling period, and a follow-up survey, respectively, conducted before, during, and after the 2023 Christmas festive season. In November 2023, 380 parents from the United Kingdom, each with at least one child under the age of 10, were recruited via the cloud-sourcing platform Prolific. They first completed a baseline survey two weeks before being invited to download the MindSampler app on November 30, 2023. This application is designed for collecting intensive longitudinal data and integrates Qualtrics surveys.

Participants received three randomly timed daily prompts between 8:00 and 20:00, from November 30, 2023 (Day 1) to January 3, 2024 (Day 35). Of those invited, 315 participants completed at least 30 daily experience sampling surveys throughout the study. On January 15, 2024, 307 of these participants also completed the follow-up survey. Participants received a total compensation of £30, distributed as follows: £4 for completing the baseline survey, £1 for installing the MindSampler app, £10 as a flat participation fee for responding to at least 30 notifications during the 35-day experience sampling period, and £5 for completing the follow-up survey. Additionally, participants who completed all three parts of the study received a £10 bonus.

After data cleaning, the final sample consisted of 293 participants, which we treated as representing distinct family units (i.e., no parent dyads), with an average age of 38.16 years (SD = 6.92). Participants were asked to indicate their gender (male, female, genderfluid/nonbinary/agender, or do not wish to say). Of the respondents, 188 identified as female and 105 as male. Additionally, 24 participants (8.11%) identified as single parents, while the remaining 269 were married or in a committed relationship. The median annual household net income fell within the range of £50,000–£59,999, which was higher than the UK’s median household income of £34,500 in 202363. On average, households had 1.86 children (SD = 0.83). A total of 62 parents (21.16%) reported having a child with special educational needs (e.g., ADHD, learning disabilities). No data on race or ethnicity was collected.
